# Role of myeloid cells in mediating the effects of lipids on ulcerative colitis

**DOI:** 10.3389/fimmu.2024.1416562

**Published:** 2024-09-02

**Authors:** Jinyin Xiao, Xiajun Guo, Keya Li, Wenpeng Luo, Youwei Lin, Wenhong Lu, Zhenquan Wang

**Affiliations:** ^1^ Department of Anorectal, the Second Affiliated Hospital of Hunan University of Traditional Chinese Medicine, Changsha, China; ^2^ Graduate School, Hunan University of Traditional Chinese Medicine, Changsha, China; ^3^ Department of Geriatric, the First People’s Hospital of Xiangtan City, Xiangtan, China

**Keywords:** Mendelian randomization, lipids, phosphatidylcholine, immune cells, myeloid cells, ulcerative colitis

## Abstract

**Objective:**

To evaluate the causal relationship between lipids and ulcerative colitis (UC) through Mendelian Randomization (MR), and to further investigate the involvement of immune cells in mediating this process.

**Methods:**

Utilizing summary statistics from genome-wide association studies (GWAS) of individuals with European ancestry, we analyzed the causal link between 179 lipid types and UC (2,569 UC cases and 453,779 controls) through Two-sample Mendelian randomization (2SMR) and Bayesian-weighted MR (BWMR). Based on this, a mediation screening of 731 immune cell phenotypes was conducted to identify exposure and mediator factors. Lastly, the role and proportion of immune cells in mediating the causal effects of lipids on UC were assessed via reverse MR (RMR) and two-step MR.

**Results:**

The results of MR showed that there was a causal relationship between the six genetically predicted lipid types and UC (P <0.05), and the four immune cell phenotypes were identified as mediators of the association between lipids and UC. Notably, Phosphatidylcholine (PC) (16:0_0:0) served as the exposure factor, and myeloid cells CD11b on CD33+ HLA DR+ CD14dim acted as the mediator. Mediation analysis showed that CD11b on CD33+ HLA DR+ CD14dim had a mediation effect of -0.0205 between PC (16:0_0:0) and UC, with the mediation effect ratio at 15.38%.

**Conclusion:**

Our findings elucidate the causal effect of lipids on UC and identify the significant mediating role of myeloid cells CD11b on CD33+ HLA DR+ CD14dim in regulating UC through PC (16:0_0:0), offering new pathways and strategies for UC clinical treatment.

## 1 Introduction

Ulcerative colitis (UC), as one of the common types of inflammatory bowel disease (IBD), is a chronic, nonspecific inflammatory condition of the gut primarily affecting the rectum and colon (1). UC is characterized by a prolonged course with frequent relapses, can manifest at any age, and is a challenging lifelong inflammatory disease that is difficult to cure (2). Surveys indicate that there are approximately 5 million UC patients globally in 2023, and its incidence rate continues to rise (3). The etiology of UC remains unclear, but research suggests a significant genetic predisposition, alongside close associations with non-genetic factors such as diet, environment, immunity and intestinal microecology (4).

In recent years, the relationship between lipid metabolism and UC pathogenesis has been increasingly pursued by researchers. Lipids, essential nutrients for the human body, mainly comprise fats, phospholipids, and sterols. They regulate energy metabolism of the body, constitute significant components of cell tissues, and play critical roles in signal transduction, so lipid abnormalities can influence the onset and progression of many diseases, including UC (5). Studies in UC patients have linked an increased incidence of the disease to reduced levels of triglycerides, total cholesterol, and low-density lipoprotein cholesterol in the blood (6, 7). A Mendelian randomization (MR) study confirmed a causal relationship between elevated high-density lipoprotein cholesterol levels and a reduced risk of IBD (8). Moreover, untreated UC patients and those in remission exhibit significant lipid level changes in the colonic mucosa compared to healthy individuals, particularly in phosphatidylcholine (PC), ceramide, and sphingomyelin (9, 10). Overall, lipid metabolism abnormalities are closely associated with UC’s development, representing important targets for diagnosis and treatment (11). However, the potential mediators in the causal relationship between lipids and UC remain unclear, warranting further investigation.

Numerous studies confirm that lipid metabolism and signal transduction play critical roles in regulating various immune cell functions and inflammation, including cholesterol, lipoproteins, Phospholipid, and steroids (12–14). Hence, we suggest that immune cells could be potential mediators through which lipids regulate UC. MR, utilizing genetic variations associated with exposure to understand the causal impact of an exposure on an outcome, can effectively test causal hypotheses in non-experimental data (15). Given the significant genetic predisposition to UC, this study aims to ascertain the causal relationship between lipids and UC via MR and further evaluate the mediating role of immune cells in lipid regulation of UC.

## 2 Methods

### 2.1 Study design

**Figure 1:**
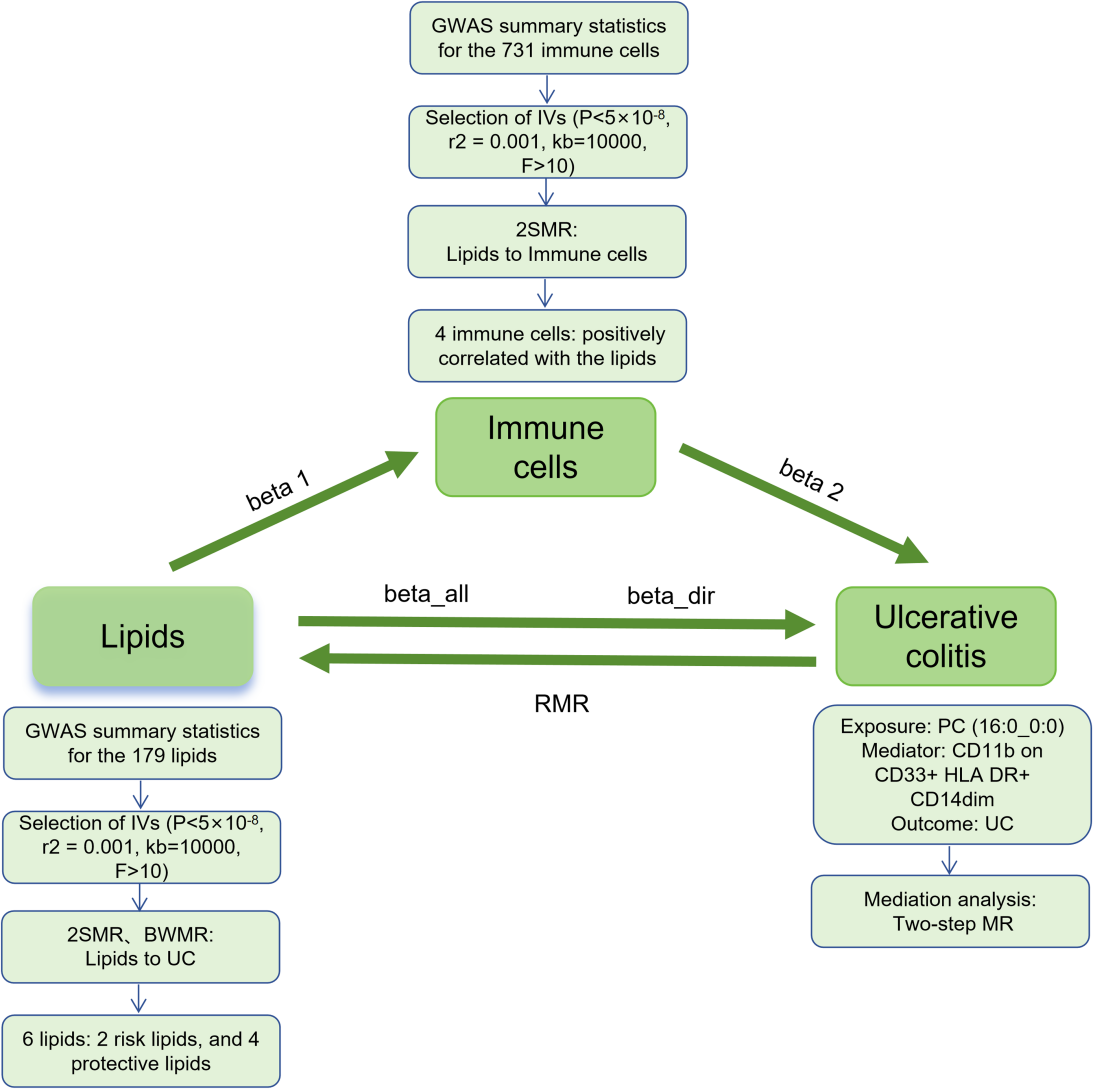
Study flow. GWAS, genome-wide association studies; IVs, instrumental variables; 2SMR, Two-sample Mendelian randomization; BWMR, Bayesian-weighted Mendelian randomization; UC, ulcerative colitis; RMR, reverse Mendelian randomization; PC, Phosphatidylcholine.

This study mainly consists of two parts (**Figure 1**). In Part 1, we used single nucleotide polymorphism (SNP) as the instrumental variables (IVs) to assess the causal relationship between 179 lipid types and UC by two-sample MR (2SMR), and further clarified the causal relationship between positive lipids and UC by Bayesian-weighted MR (BWMR). In Part 2, we screened 731 immunophenotypes for potential immune cell mediation candidates between lipid and UC, and calculated the mediation effect and proportion of one of the immune cell phenotype mediating lipid regulation of UC.

### 2.2 Data sources for exposure, outcome, and mediator

In this study, data for exposure, outcome, and mediator were all derived from participants of genome-wide association studies (GWAS) with European ancestry. We declare that all data used in the course of this study were publicly available, did not involve personal privacy and information, and could be located in the corresponding articles or platform databases. The related research has been approved by institutional review boards, hence, ethical review was not required for this study.

The GWAS summary statistics for 179 serum lipids were sourced from the GWAS catalog (https://www.ebi.ac.uk/gwas) (GCST90277238-GCST90277416), compiling GWAS data for 179 lipid species across 13 lipid categories from 7,174 Finnish individuals (16). The GWAS summary statistics for 731 immune cell phenotypes were publicly obtained from the GWAS catalog (GCST90001391 to GCST90002121), summarizing statistics for 731 immune cell phenotype features from a cohort of 3,757 Europeans, including relative count (RC), absolute count (AC), median fluorescence intensity (MFI) reflecting surface antigen levels, and morphological parameters (MP) (17). The UC data were drawn from the GWAS catalog’s summary data source (GCST90044155), which included 456348 individuals of European ancestry, including 2569 UC cases and 453779 controls (18). UC cases were identified using the “PheCode 555.2” classification, which is a disease categorization tool based on electronic health record data and the International Classification of Diseases (ICD codes). For detailed information on participant characteristics, genotyping, imputation, and quality control, please refer to the GWAS catalog website (https://www.ebi.ac.uk/gwas).

### 2.3 Selection of IVs

Initially, we selected SNPs that met the genome-wide significance threshold (P < 5×10^-8^). To prevent linkage disequilibrium (LD) from affecting the analysis of subsequent results, we set the parameters to r^2^ = 0.001 and kb = 10,000, meaning SNPs with r^2^ > 0.001 and within a 10,000 kb range were excluded. Finally, to mitigate the bias in causal inference due to weak instrumental variables, we used an F-statistic < 10 as the criterion for excluding weak instruments (F-statistic = β^2^/SE^2^, where β is the allele effect size, and SE is the standard error) (19).

### 2.4 Statistical analysis

The MR analysis in this study was performed using the “TwoSampleMR” package (version 0.5.11) within the R software version 4.3.3. Five common MR statistical methods were employed: MR Egger, Weighted median, Inverse Variance Weighted (IVW), Simple mode, and Weighted mode, with IVW being the primary analytical approach. Sensitivity analyses included tests for heterogeneity, pleiotropy, and leave-one-out analysis. Heterogeneity among IVs was assessed using IVW and MR-Egger tests, with p-values <0.05 indicating the presence of heterogeneity in the study. Pleiotropy was detected with the MR-Egger intercept test, and robustness of the results was evaluated, with p-values <0.05 indicating the pleiotropy. The leave-one-out sensitivity analysis was used to assess whether a single SNP had an excessive effect on the overall MR estimate (15, 20). Furthermore, BWMR and reverse MR (RMR) were performed to clarify the causal relationship between lipids and UC, as well as the potential presence of reverse causality. Finally, a two-step MR was used to calculate the mediating effects and mediation proportion of immune cells on the relationship between lipids and UC. In the two-step MR, the first step assessed the causal effect of lipids on immune cells to obtain beta 1; the second step assessed the effect of immune cells on UC to obtain beta 2. The mediating effect (beta12) = beta 1 × beta 2, the direct effect (beta_dir) = total effect (beta_all) - beta12; and the mediation proportion = beta12/beta_all (**Figure 1**).

## 3 Results

### 3.1 Causal effects of lipids on UC

**Figure 2:**
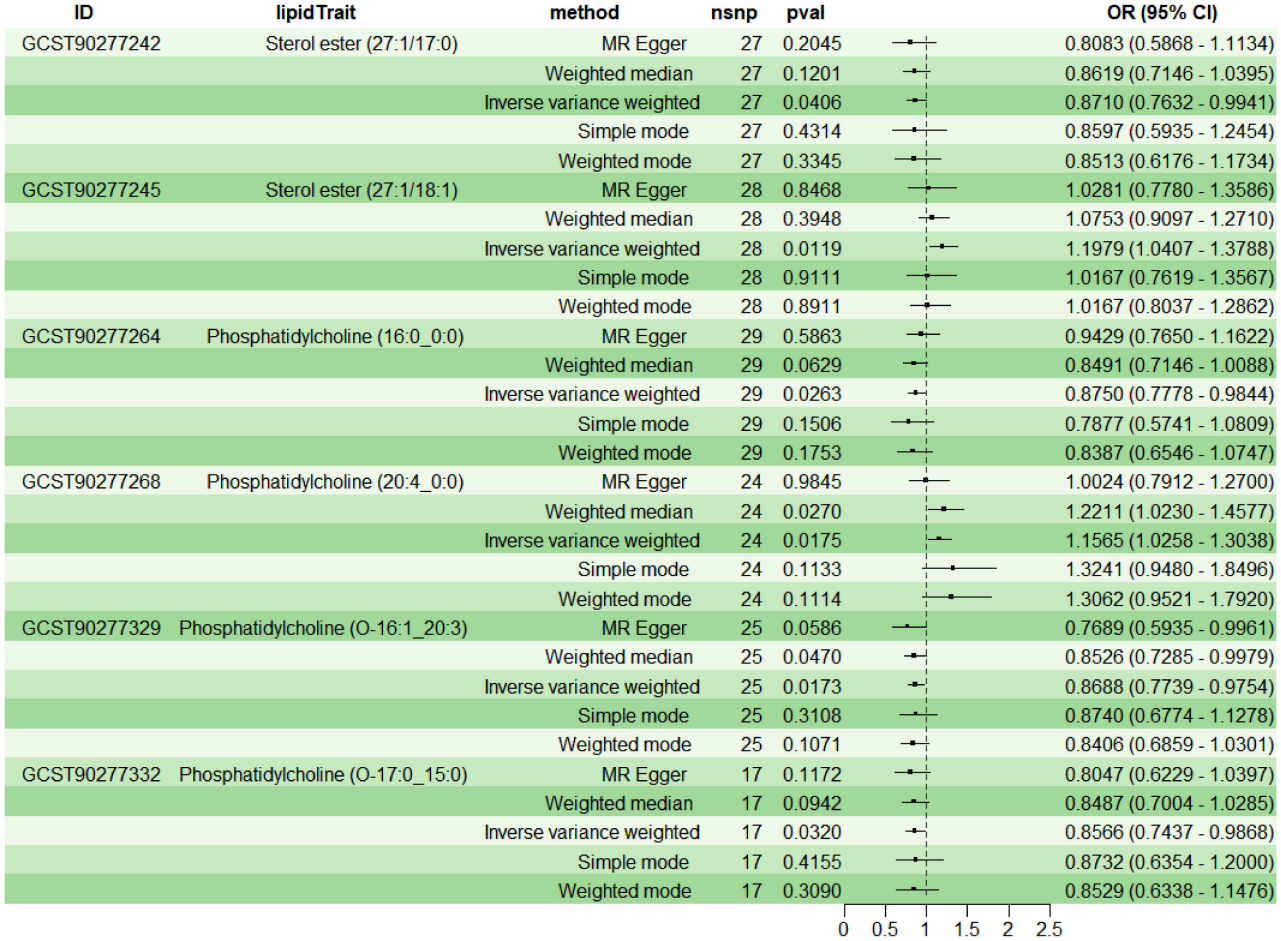
2SMR analysis of the causal relationship between six lipids and UC.

**Figure 3:**
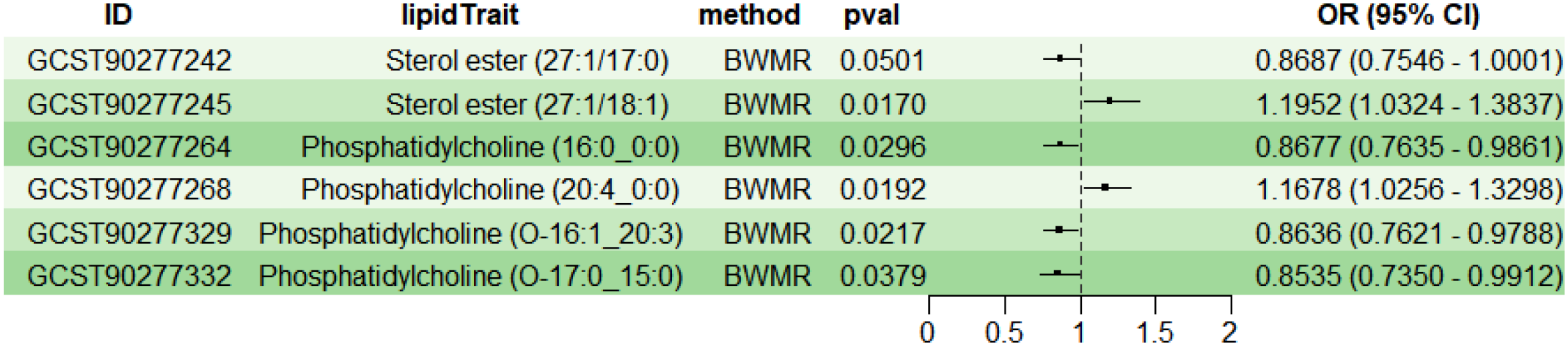
BWMR analysis of the causal relationships between six lipids and UC. BWMR, Bayesian-weighted Mendelian randomization.

To explore the causal effects of 179 serum lipids on UC, this study utilized 2SMR analysis with IVW as the main analytical method. After filtering the GWAS data for 179 serum lipids, removing those affected by linkage disequilibrium and weak instrumental variables, six significant lipid types were identified (P<5×10^-8^) (**Figure 2**). Among these, two lipid types were identified as risk factors for the development of UC (OR>1, P<0.05), namely Sterol ester (27:1/18:1) and PC (20:4_0:0); while four lipid types were protective factors against UC (OR<1, P<0.05), specifically Sterol ester (27:1/17:0), PC (16:0_0:0), PC (O-16:1_20:3), and PC (O-17:0_15:0). Sensitivity analysis indicated negligible heterogeneity (P>0.05) and horizontal pleiotropy (P>0.05) among these six lipid types, substantiating a robust and credible causal relationship between lipids and UC (**Supplementary Table 1**). Leave-one-out sensitivity analysis and funnel plots validated the effectiveness and non-heterogeneity of the MR results (**Supplementary Figure File 1**). Finally, through BWMR, a clear causal relationship between these six lipid types and UC was further confirmed, aligning with the initial 2SMR results (**Figure 3**).

### 3.2 Impact of lipids on immune cells

**Figure 4:**
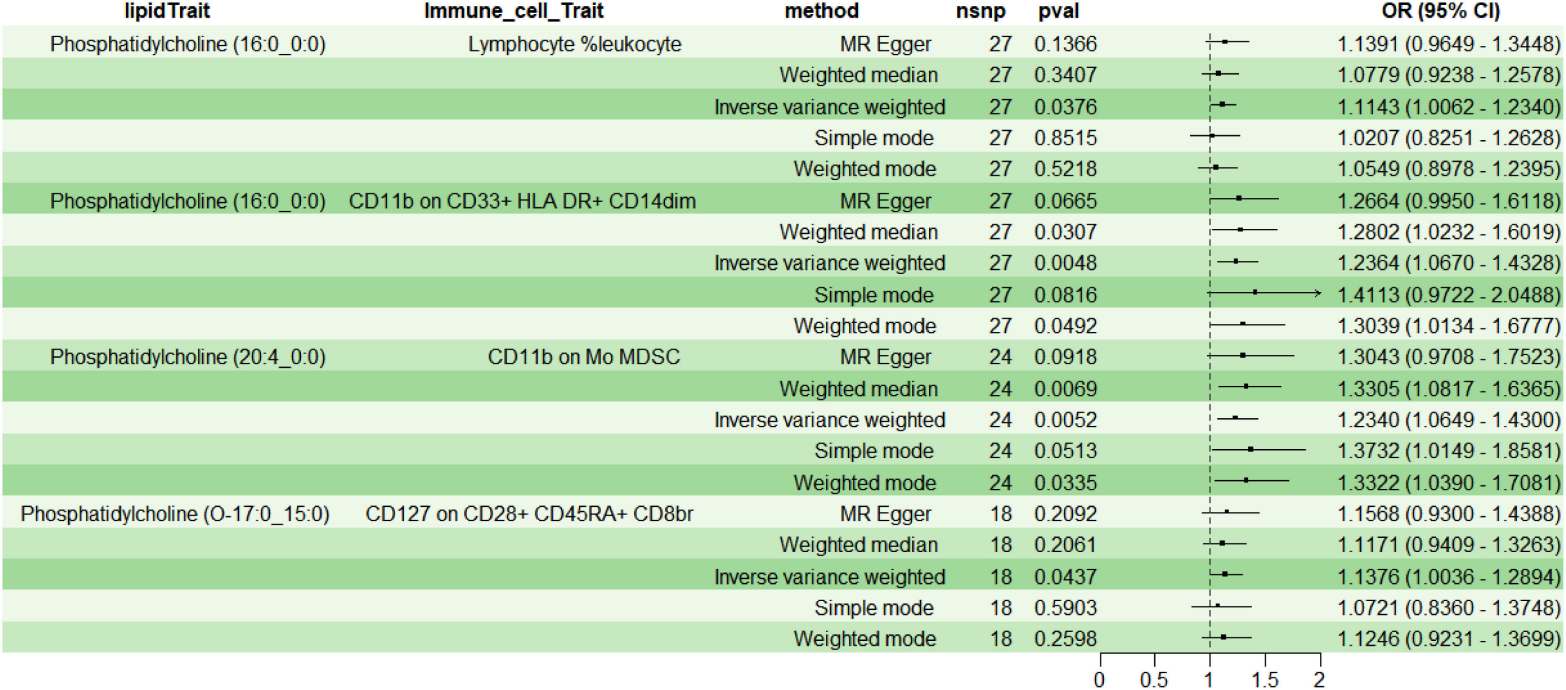
Causal relationship between lipids and immune cells.

Employing 2SMR with IVW as the primary method, four immune cell phenotypes were screened from 731 potential immune mediators, meeting the selection criteria: TBNK Panel: Lymphocyte %leukocyte (RC); Myeloid cell Panel: CD11b on CD33+ HLA DR+ CD14dim (MFI) and CD11b on Mo MDSC (MFI); Treg Panel: CD127 on CD28+ CD45RA+ CD8br (MFI). The results indicated a positive correlation between above four immune cell phenotypes and lipids (OR>1, P<0.05) (**Figure 4**). Additionally, sensitivity analysis indicated no heterogeneity (P>0.05) or pleiotropy (P>0.05) for the immune cell phenotypes, affirming the robust and reliable causal relationship between lipids and immune cells (**Supplementary Table 2**); leave-one-out sensitivity analysis and funnel plots demonstrated the validity and non-heterogeneity of the MR results (**Supplementary Figure File 2**). Based on the above MR findings, lipid PC (16:0_0:0) was selected as the exposure factor, and myeloid cell CD11b on CD33+ HLA DR+ CD14dim was selected as the mediator for subsequent mediation MR analysis.

### 3.3 RMR

**Figure 5:**
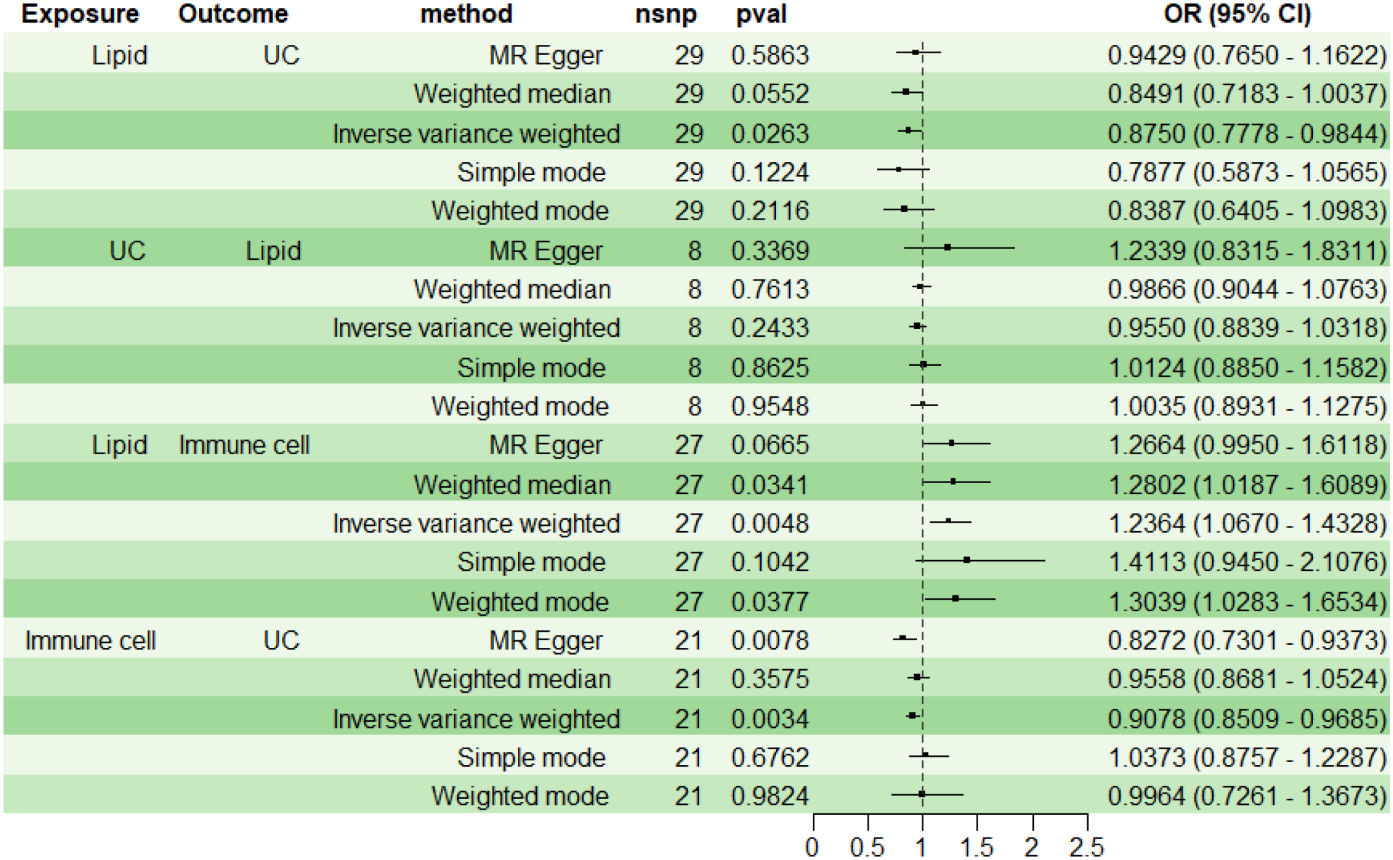
Immune Cell Mediation of the Causal Effect of Lipid on UC. UC, ulcerative colitis.

For subsequent mediation analysis, we used RMR to exclude the reverse causality of UC on lipid PC (16:0_0:0) with IVW as the main analysis method. The results showed that there was no reverse causality between UC and PC (16:0_0:0) (P> 0.05) (**Figure 5**).

### 3.4 Immune cell mediation of the causal effect of lipid on UC

Using two-step MR with IVW as the primary analytical method, we assessed the mediation effect and proportion mediated by the immune cell phenotype CD11b on CD33+ HLA DR+ CD14dim in the causal relationship between lipid PC (16:0_0:0) and UC. The findings indicate a causal relationship between PC (16:0_0:0) and UC, with PC (16:0_0:0) acting as a protective factor for UC (OR<1, P<0.05). A positive correlation was observed between PC (16:0_0:0) and CD11b on CD33+ HLA DR+ CD14dim (OR>1, P<0.05), while CD11b on CD33+ HLA DR+ CD14dim was negatively correlated with UC (OR<1, P<0.05) (**Figure 5**). The mediation effect of myeloid cell CD11b on CD33+ HLA DR+ CD14dim between PC (16:0_0:0) and UC was -0.0205, with a total effect of -0.1335, a direct effect of -0.1130, and a mediation proportion of 15.38%.

## 4 Discussion

Our study provides new evidence for the causal effects of lipids on UC, confirming the causal relationships between six serum lipid types and UC via 2SMR, identifying Sterol ester (27:1/18:1) and PC (20:4_0:0) as risk factors for UC development, while Sterol ester (27:1/17:0), PC (16:0_0:0), PC (O-16:1_20:3), and PC (O-17:0_15:0) as protective factors. Building on this foundation, validation with BWMR reaffirmed the definitive causal relationships between the six lipids and UC, proving the reliability of these findings. Moreover, we investigated potential immune mediators in the pathway from lipid to UC and identified four eligible immune cell phenotypes. The results showed positive correlations between lipids and the following immune cell: TBNK Panel: Lymphocyte %leukocyte; Myeloid cell Panel: CD11b on CD33+ HLA DR+ CD14dim and CD11b on Mo MDSC; Treg Panel: CD127 on CD28+ CD45RA+ CD8br. Based on the MR results, we finally included PC (16:0_0:0) as the exposure factor and CD11b on CD33 + HLA DR + CD14dim as the mediating factor. RMR confirmed the lack of reverse causality between UC and PC (16:0_0:0), leading us to explore the mediation effect of CD11b on CD33+ HLA DR+ CD14dim on PC (16:0_0:0) in regulating UC. The results indicated a mediation effect of -0.0205 and a proportion mediated of 15.38%. These findings reveal the protective role of PC (16:0_0:0) in UC, and that myeloid cell CD11b on CD33+ HLA DR+ CD14dim plays a significant mediating role in the regulation of UC by PC (16:0_0:0).

Reduced PC content in colonic mucus is a significant characteristic of UC patients (21). Studies have identified PC as crucial for maintaining cellular membrane integrity and mediating cholesterol transport, and PC can be secreted by enterocytes, which is the main phospholipid of intestinal mucus and is crucial for maintaining the intestinal mucosal barrier, so PC is considered a key factor in regulating the pathogenesis of UC (22–24). The study found significant lipid dysregulation in UC patients serum and mouse colon, particularly marked decreases in triglycerides and PC (25). Further, it was discovered that exogenous PC 34:1 can significantly alleviate symptoms in UC mice, reducing intestinal tissue damage and increasing colonic mucus layer thickness, thereby exerting an anti-UC effect. This mechanism is associated with the inhibition of glutamate conversion to N-acetylglutamate, thereby enhancing fumarate levels (25). Dimethyl fumarate derived from fumarate has been demonstrated to exert immunoregulatory, anti-inflammatory and antioxidant effects to treat intestinal diseases like IBD by regulating the Kelch-like ECH-associated protein 1 (Keap1)/NF-E2-Related Factor 2 (Nrf2), NOD-like receptor family pyrin domain containing 3 (NLRP3), hypoxia inducible factor-1 (HIF-1α) and nuclear factor kappa-B (NF-κB) signaling pathways (26–28). These findings underscore PC’s vital role in maintaining the intestinal mucosal barrier and mitigating inflammation, suggesting PC deficiency as a crucial factor in UC development. The potential therapeutic value of PC in UC treatment has spurred research and development of novel clinical drugs, with numerous trials indicating that delayed-release phosphatidylcholine can effectively address PC deficiency in UC patients, alleviating the condition (29–31).

Although PC demonstrates significant immunomodulatory and anti-inflammatory effects (32–34), there is limited research on whether immune cells mediate the process of PC regulating UC. Myeloid cells, as the main cellular components of the innate immune response, play a crucial role in the immune system, which mainly include such as macrophages, dendritic cells, monocytes and granulocytes (35). Physiologically, as an essential component of the cell membrane, PC is crucial for maintaining the integrity of myeloid cell membranes (23). Moreover, PC acts as a signaling molecule for myeloid cells like macrophages and neutrophils, thus influencing inflammation and immune responses. For instance, David et al. found that negatively charged membrane phospholipids could control innate immune responses to infections by regulating macrophage metabolic reprogramming and activating downstream signaling cascades (36). Additionally, PC is involved in protecting macrophages from palmitate-induced endoplasmic reticulum stress and pro-inflammatory activation (37). As a precursor of PC, choline has been shown to regulate the activation of the NLRP3 inflammasome and the production of interleukin (IL)-1β and IL-18 in macrophages through its metabolism (38). The activation of the NLRP3 signaling pathway is considered a significant cause of UC pathogenesis, hence inhibiting this pathway is seen as a crucial strategy for UC treatment (39). Furthermore, oxidized PC also regulates the formation of neutrophil extracellular traps (NETs) (40), which are critical to the pathogenesis of UC (41, 42).

While current studies lack direct evidence of myeloid cells mediating PC’s protective role in UC, existing experimental research indirectly supports the possibility of such mediation. To date, we are the first to rigorously determine the causal effect between myeloid cell-mediated PC and UC risk using various MR methods, providing new evidence and mechanisms for the clinical treatment of UC with PC. However, our study is limited to European populations and may not represent all ethnicities. Additionally, due to the extensive work involved, our research did not further explore the causal effects of other lipids and immune cell phenotypes, which may be addressed in future work. Finally, the lack of animal or clinical level evidence to support the role of myeloid cells in mediating PC protection against UC will be the focus of our future research efforts.

## 5 Conclusion

In conclusion, our multi-MR analysis confirmed the causal effect between PC (16:0_0:0) and UC, and identified the mediating role of myeloid cells CD11b on CD33+ HLA-DR+ CD14dim in this process. Our study extends the understanding of PC’s role in UC to the immune cells, providing new pathways and theoretical support for the clinical use of PC in treating UC.

## Data Availability

The original contributions presented in the study are included in the article/**Supplementary Material**. Further inquiries can be directed to the corresponding author.
