# Supplementary material for: Role of myeloid cells in mediating the effects of lipids on ulcerative colitis
Source: Front Immunol. 2024 Sep 2;15:1416562. doi: 10.3389/fimmu.2024.1416562 (PMC11402659; doi:10.3389/fimmu.2024.1416562)
Supplement: Supplementary file 1 [file DataSheet1.docx]

Supplementary Material

Role of myeloid cells in mediating the effects of lipids on ulcerative colitis

Jinyin Xiao^1,2^ ^†^, Xiajun Guo^3^ ^†^, Keya Li^1^, Youwei Lin^2^, Wenhong Lu^1^, Zhenquan Wang^1*^, Wenpeng Luo^1*^

*** Correspondence:** Zhenquan Wang: [320035@hnucm.edu.cn](mailto:320035@hnucm.edu.cn); Wenpeng Luo: 320044@hnucm.edu.cn

# Supplementary Figures and Tables

## Supplementary Figure File 1


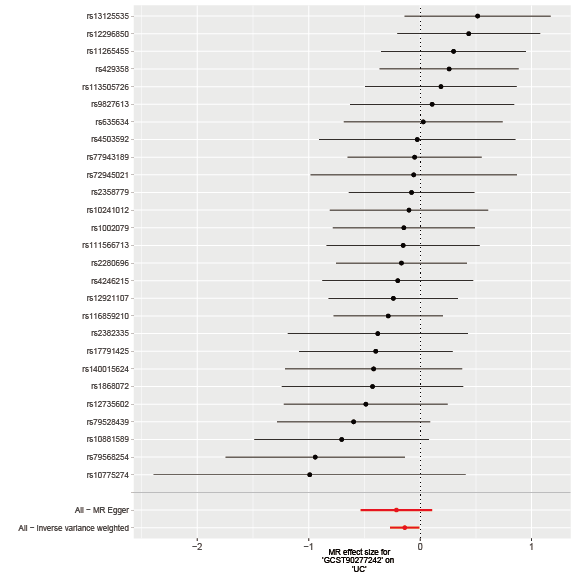
 Supplementary Figure 1A. Forest plot (GCST90277242-UC)


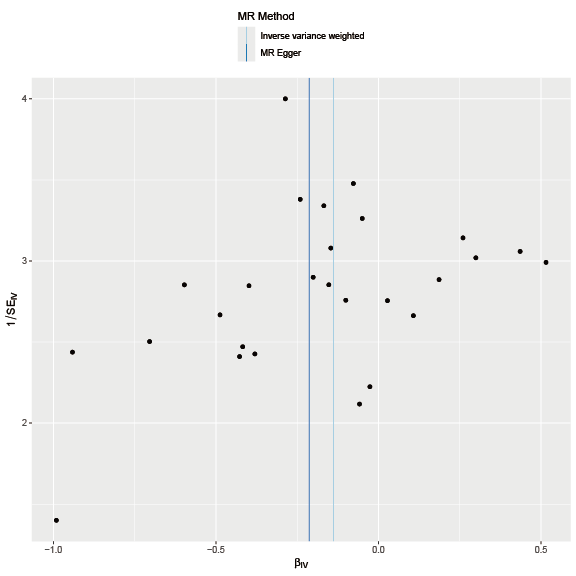


Supplementary Figure 1B. Funnel plot (GCST90277242-UC)


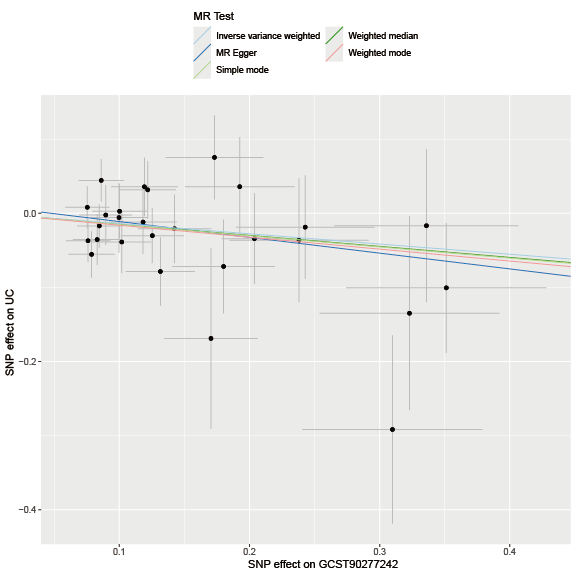


Supplementary Figure 1C. Scatter plot (GCST90277242-UC)


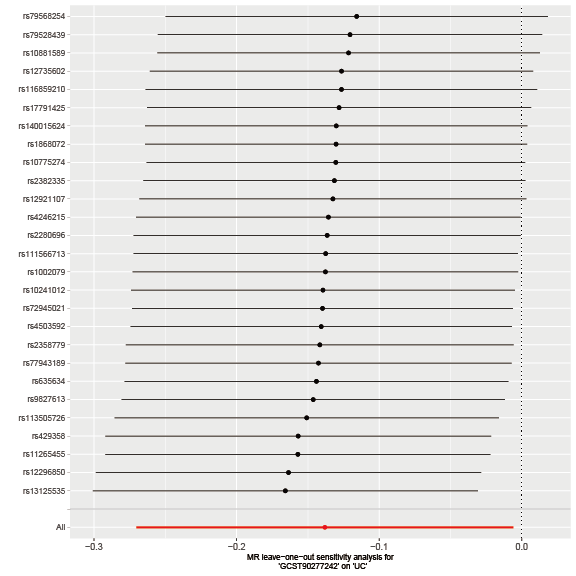


Supplementary Figure 1D. Sensitivity-analysis (GCST90277242-UC)


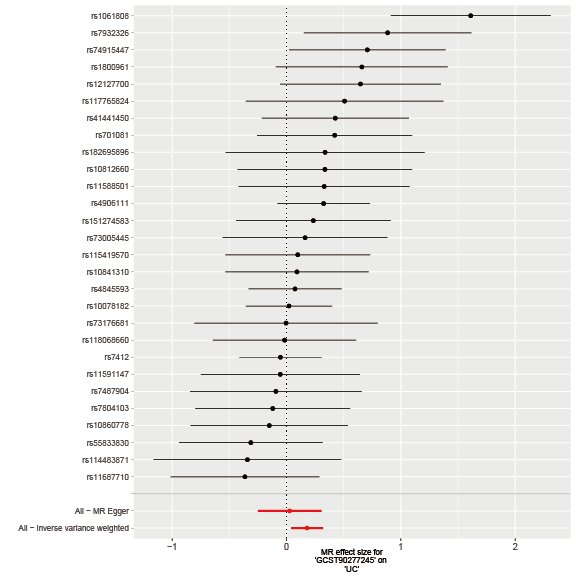


Supplementary Figure 2A. Forest plot (GCST90277245-UC)


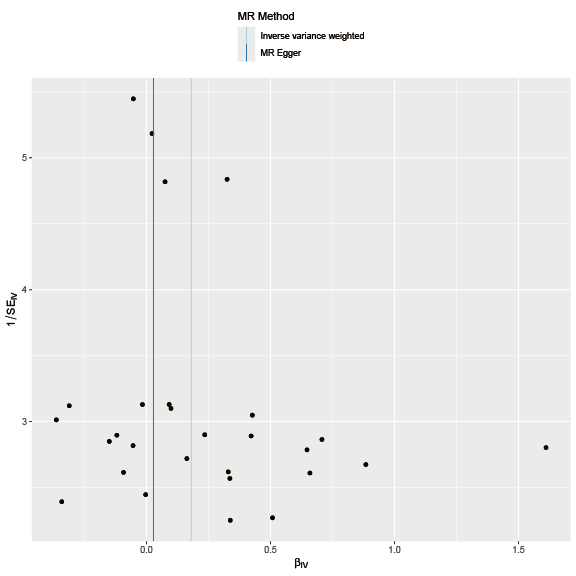
 Supplementary Figure 2B. Funnel plot (GCST90277245-UC)


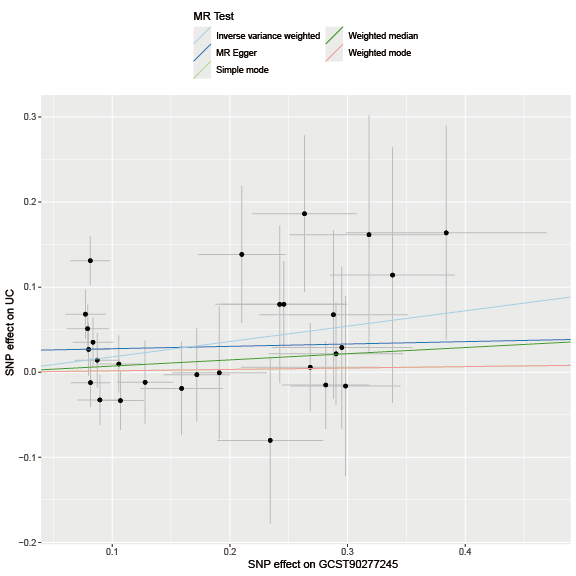
 Supplementary Figure 2C. Scatter plot (GCST90277245-UC)


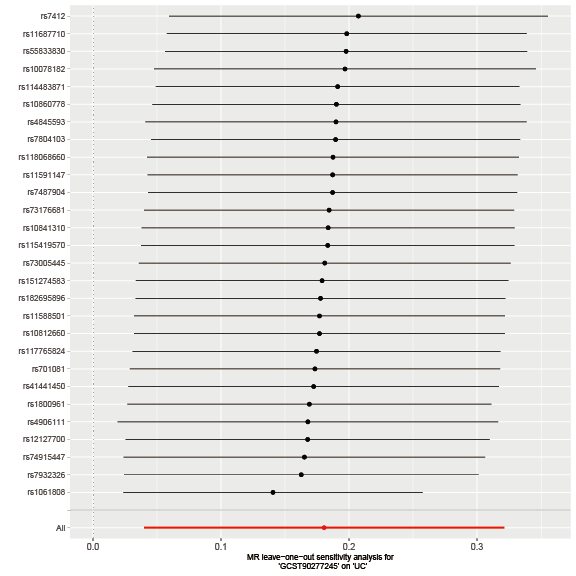


Supplementary Figure 2D. Sensitivity-analysis (GCST90277245-UC)


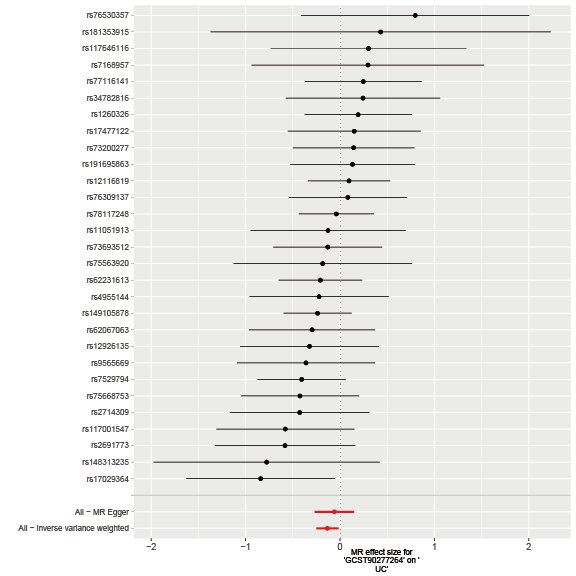
 Supplementary Figure 3A. Forest plot (GCST90277264-UC)


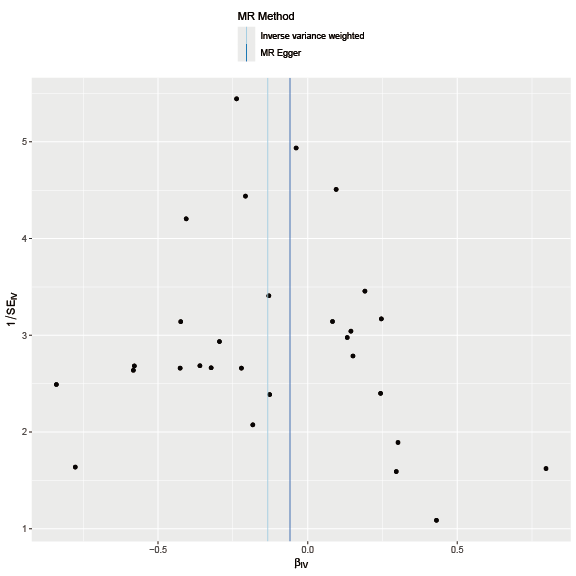
 Supplementary Figure 3B. Funnel plot (GCST90277264-UC)


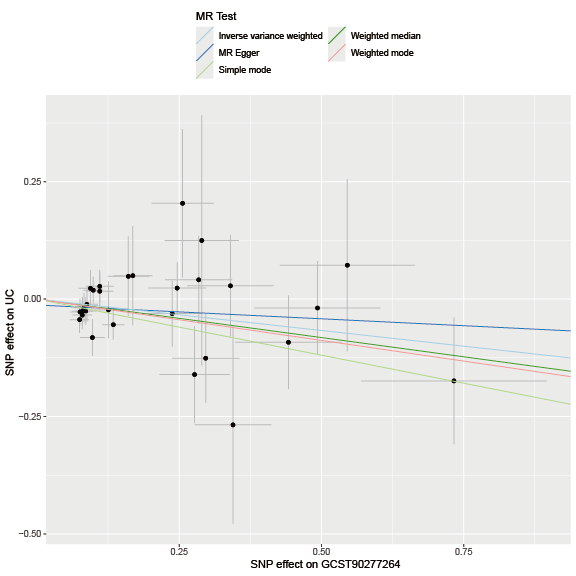
 Supplementary Figure 3C. Scatter plot (GCST90277264-UC)
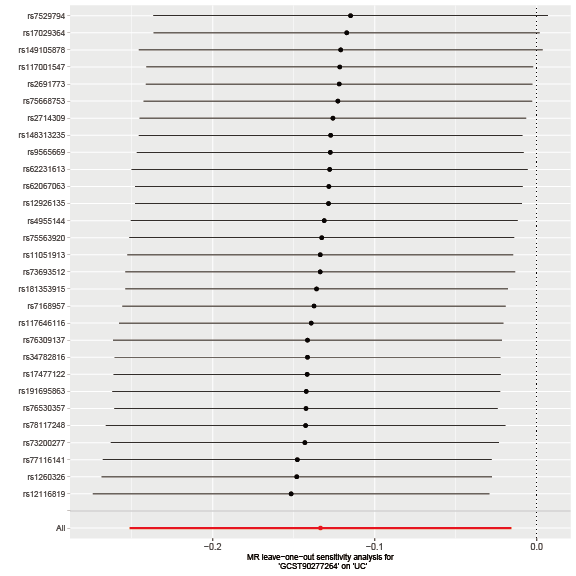
 Supplementary Figure 3D. Sensitivity-analysis (GCST90277264-UC)


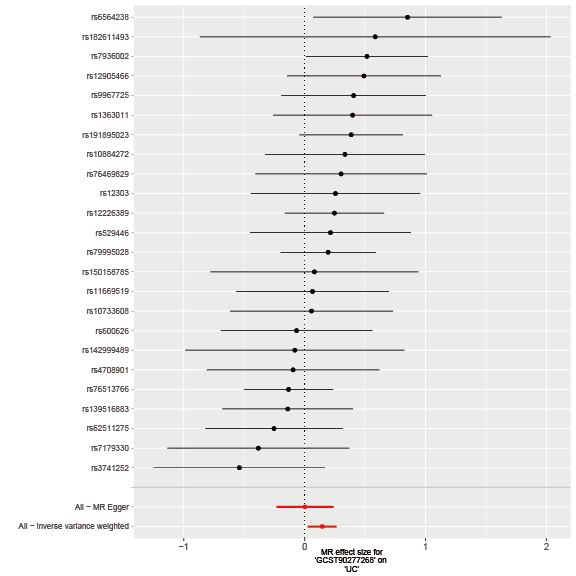
 Supplementary Figure 4A. Forest plot (GCST90277268-UC)
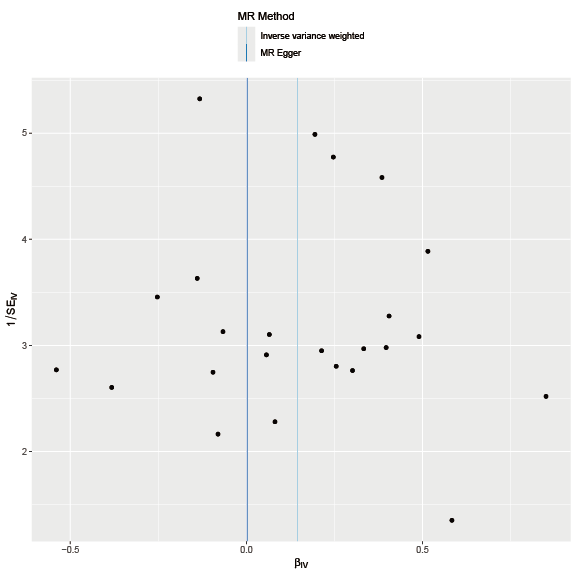
 Supplementary Figure 4B. Funnel plot (GCST90277268-UC)
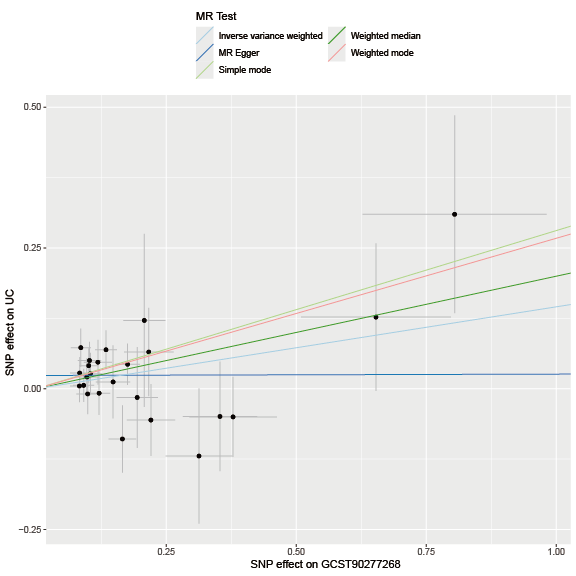
 Supplementary Figure 4C. Scatter plot (GCST90277268-UC)
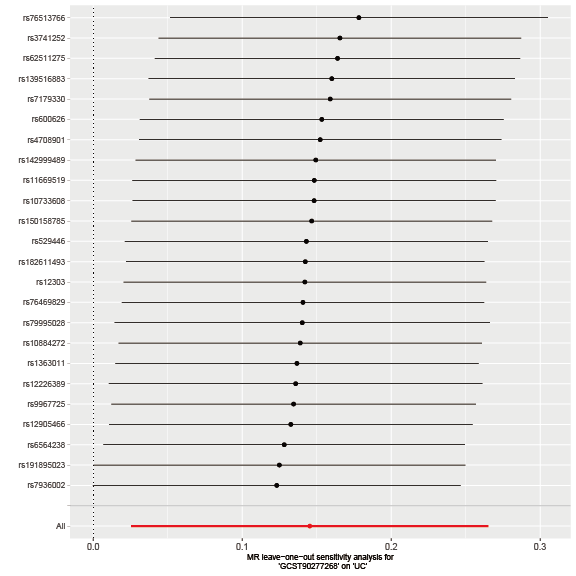
 Supplementary Figure 4D. Sensitivity-analysis (GCST90277268-UC)


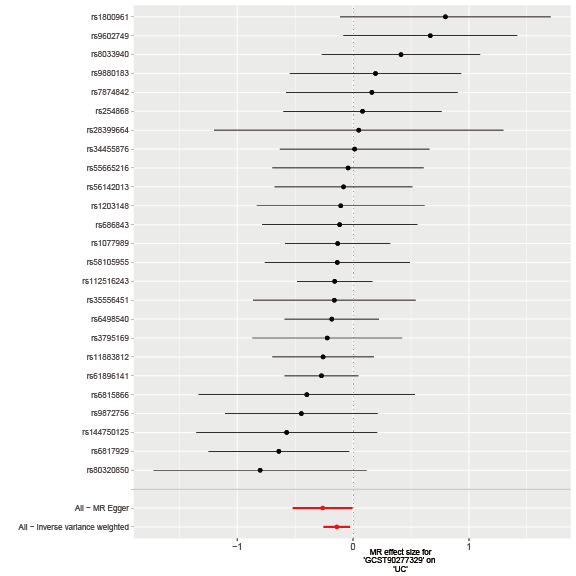
 Supplementary Figure 5A. Forest plot (GCST90277329-UC)
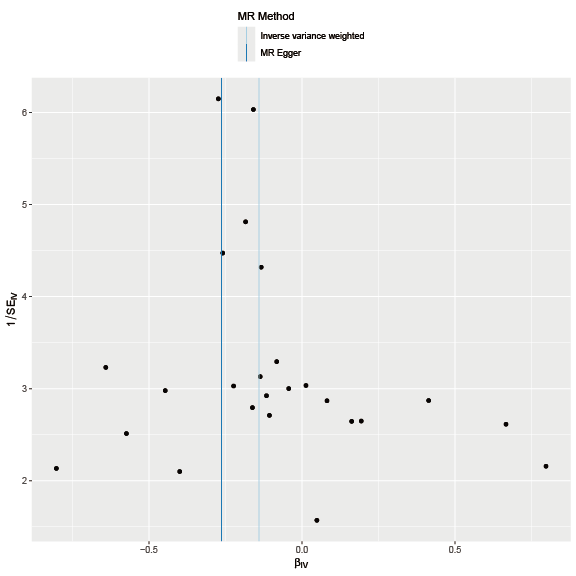
 Supplementary Figure 5B. Funnel plot (GCST90277329-UC)
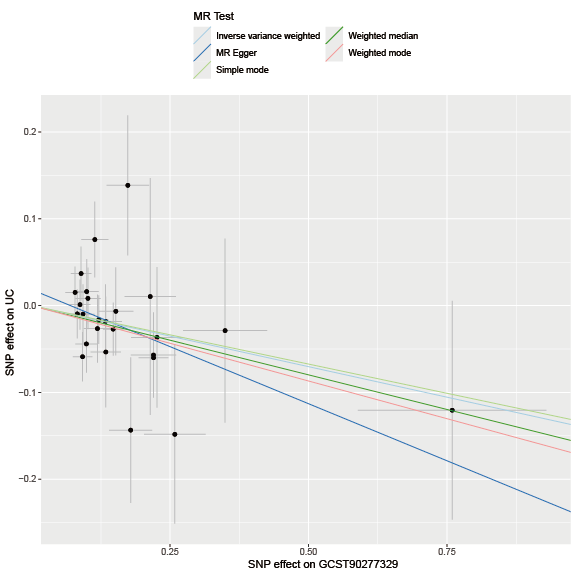
 Supplementary Figure 5C. Scatter plot (GCST90277329-UC)
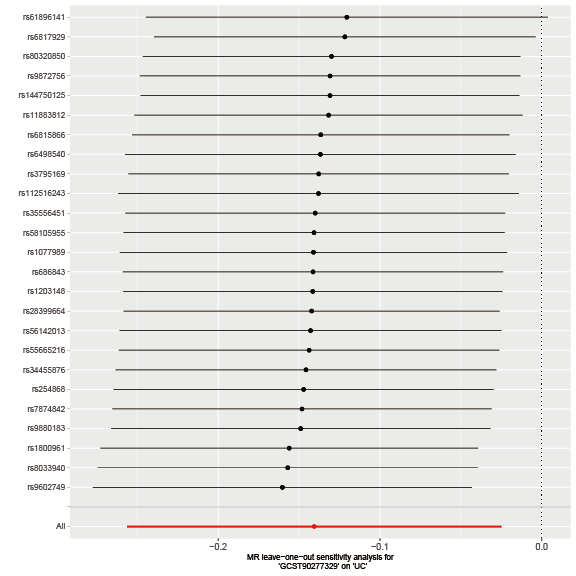
 Supplementary Figure 5D. Sensitivity-analysis (GCST90277329-UC)


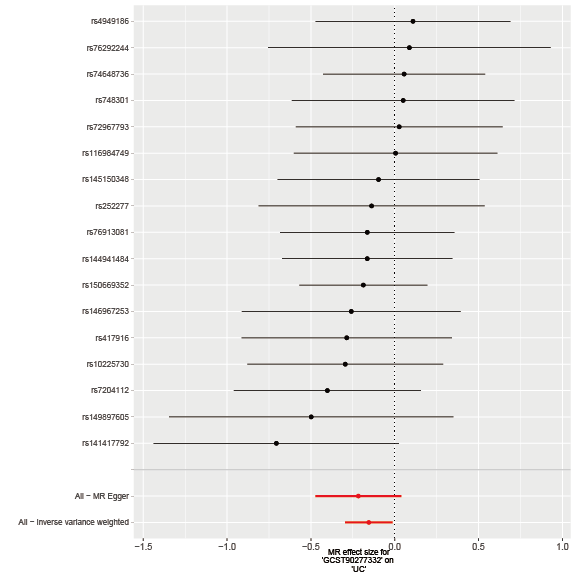
 Supplementary Figure 6A. Forest plot (GCST90277332-UC)
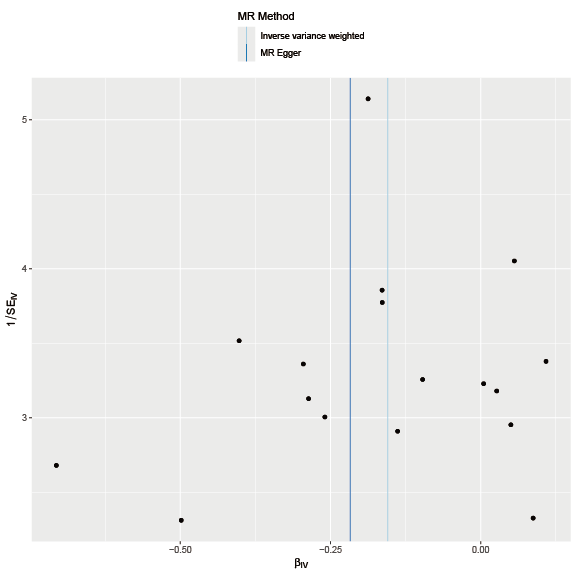
 Supplementary Figure 6B. Funnel plot (GCST90277332-UC)
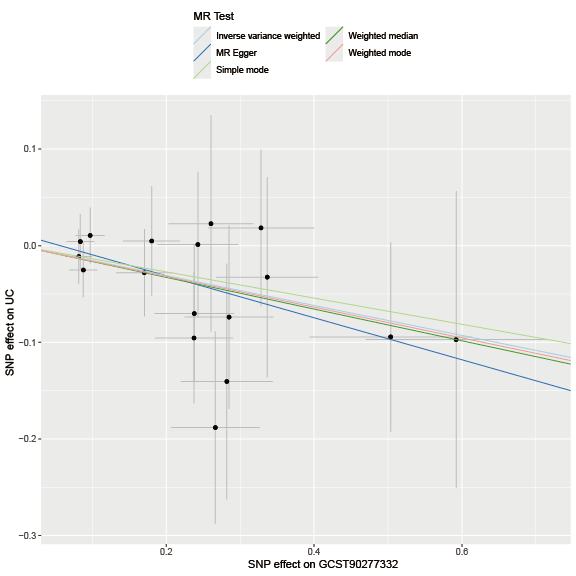
 Supplementary Figure 6C. Scatter plot (GCST90277332-UC)


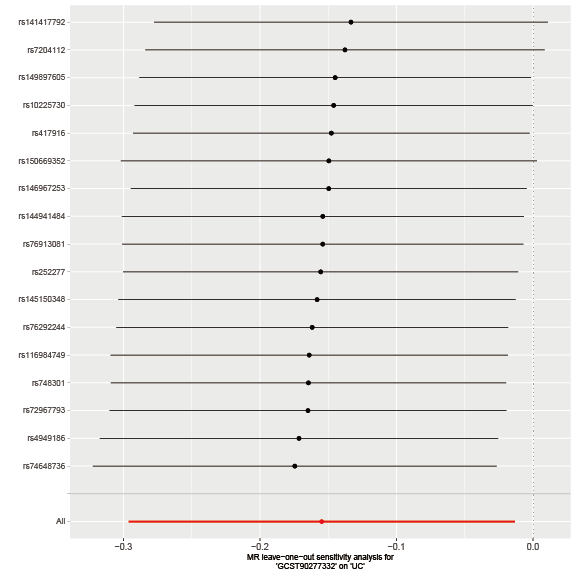
 Supplementary Figure 6D. Sensitivity-analysis (GCST90277332-UC)

## Supplementary Figure File 2


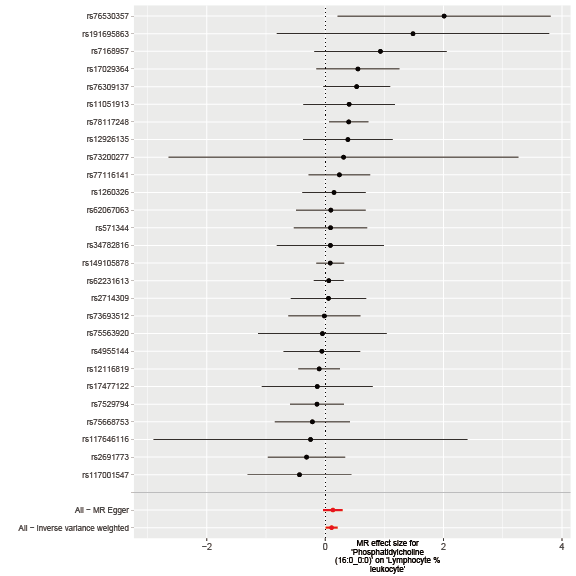
 Supplementary Figure 1A. Forest plot (Phosphatidylcholine (16:0_0:0)-Lymphocyte %leukocyte)
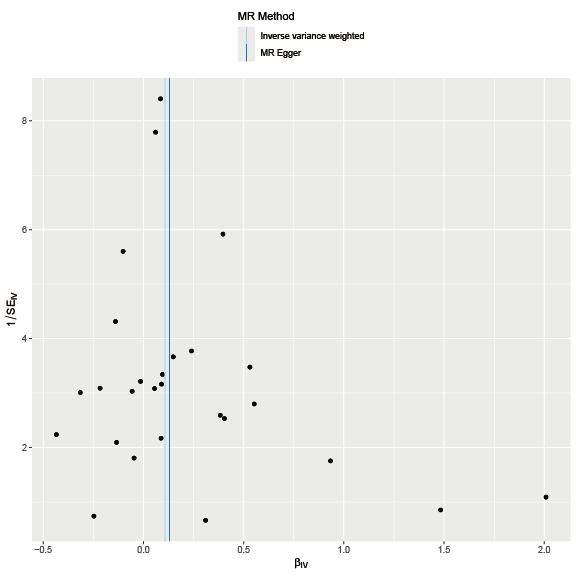
 Supplementary Figure 1B. Funnel plot (Phosphatidylcholine (16:0_0:0)-Lymphocyte %leukocyte)
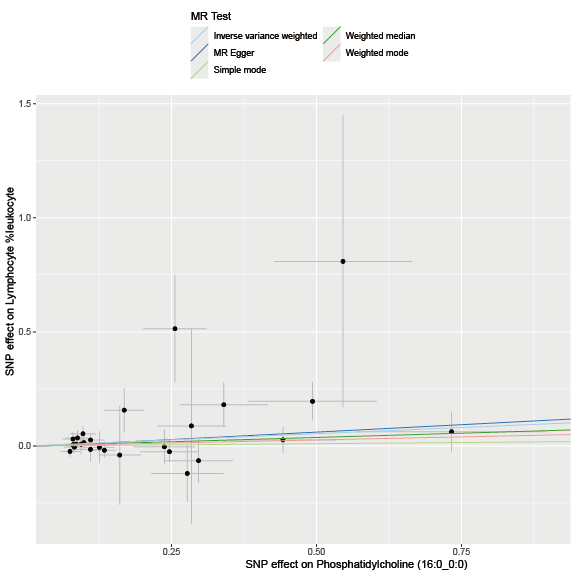
 Supplementary Figure 1C. Scatter plot (Phosphatidylcholine (16:0_0:0)-Lymphocyte %leukocyte)
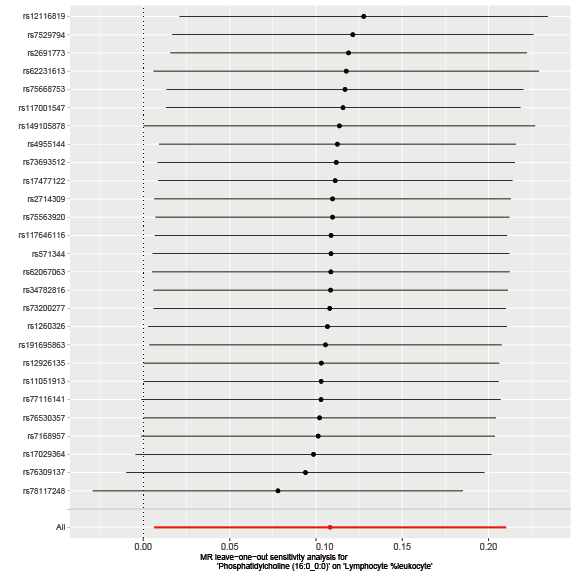
 Supplementary Figure 1D. Sensitivity-analysis (Phosphatidylcholine (16:0_0:0)-Lymphocyte %leukocyte)


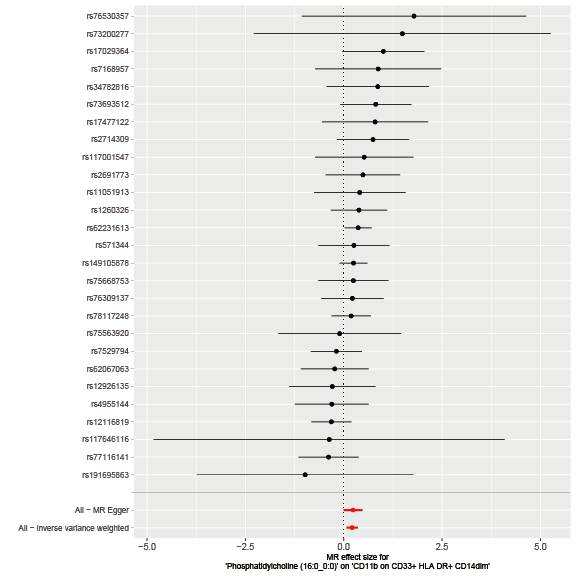
 Supplementary Figure 2A. Forest plot (Phosphatidylcholine (16:0_0:0)-CD11b on CD33+ HLA DR+ CD14dim)
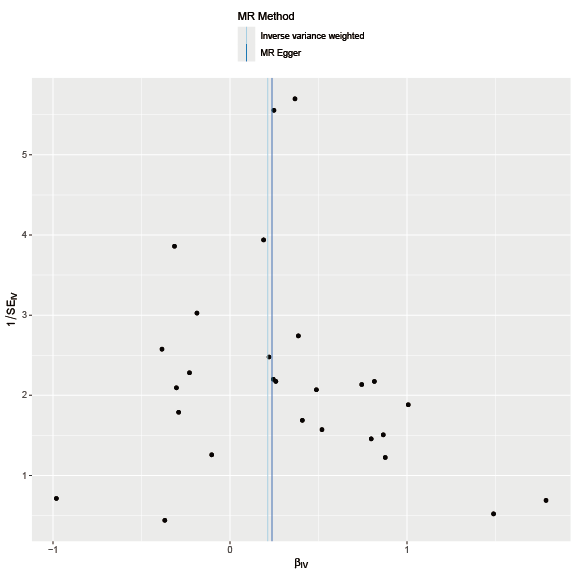
 Supplementary Figure 2B. Funnel plot (Phosphatidylcholine (16:0_0:0)-CD11b on CD33+ HLA DR+ CD14dim)
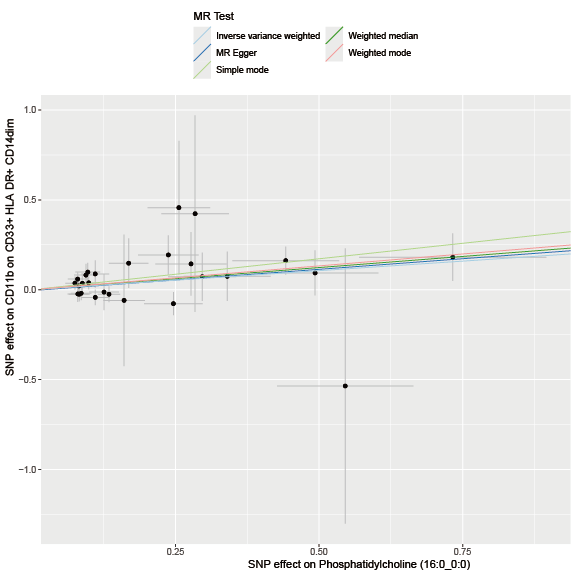
 Supplementary Figure 2C. Scatter plot (Phosphatidylcholine (16:0_0:0)- CD11b on CD33+ HLA DR+ CD14dim)
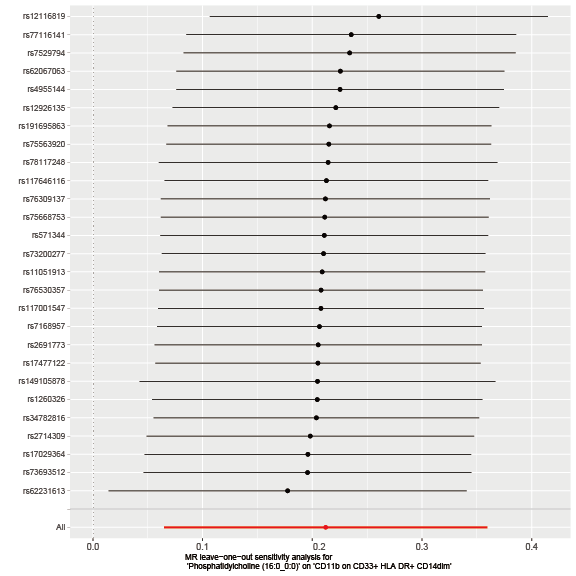
 Supplementary Figure 2D. Sensitivity-analysis (Phosphatidylcholine (16:0_0:0)-CD11b on CD33+ HLA DR+ CD14dim)


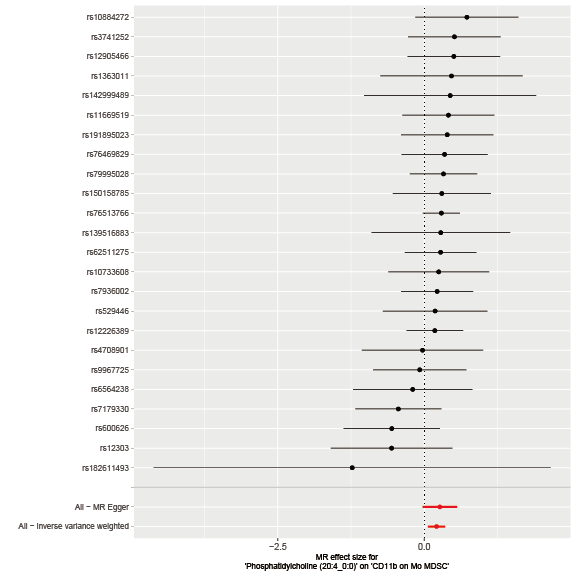
 Supplementary Figure 3A. Forest plot (Phosphatidylcholine (20:4_0:0)-CD11b on Mo MDSC)
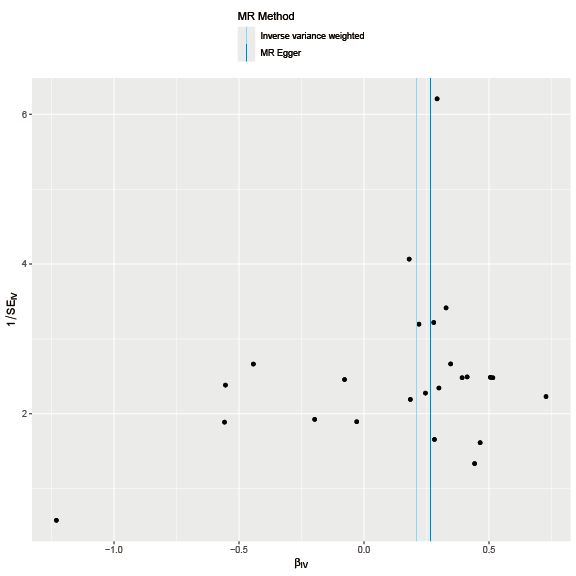
 Supplementary Figure 3B. Funnel plot (Phosphatidylcholine (20:4_0:0)-CD11b on Mo MDSC)
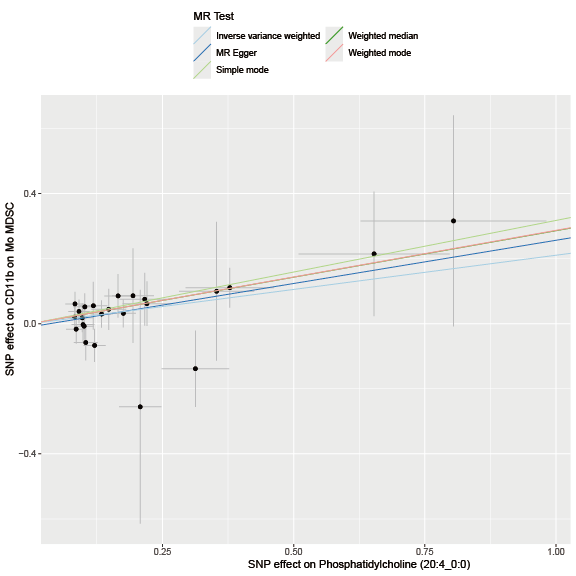
 Supplementary Figure 3C. Scatter plot (Phosphatidylcholine (20:4_0:0)-CD11b on Mo MDSC)
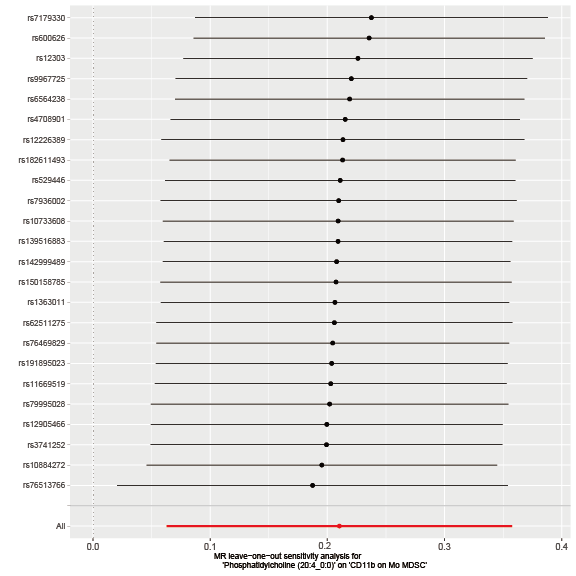
 Supplementary Figure 3D. Sensitivity-analysis (Phosphatidylcholine (20:4_0:0)-CD11b on Mo MDSC)


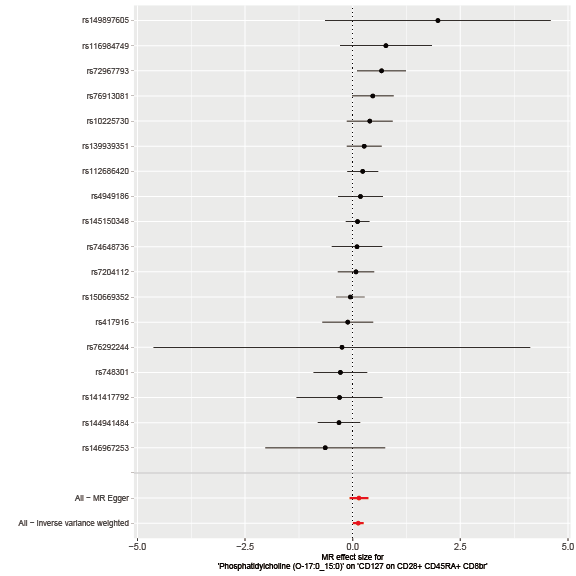
 Supplementary Figure 4A. Forest plot (Phosphatidylcholine (O-17:0_15:0)-CD127 on CD28+ CD45RA+ CD8br)
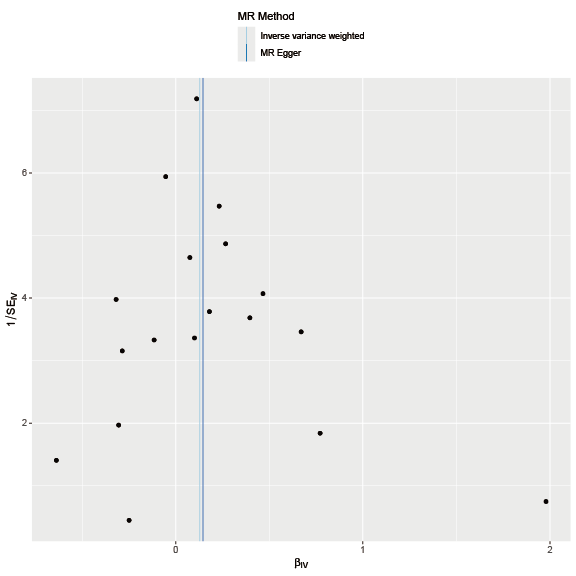
 Supplementary Figure 4B. Funnel plot (Phosphatidylcholine (O-17:0_15:0)-CD127 on CD28+ CD45RA+ CD8br)
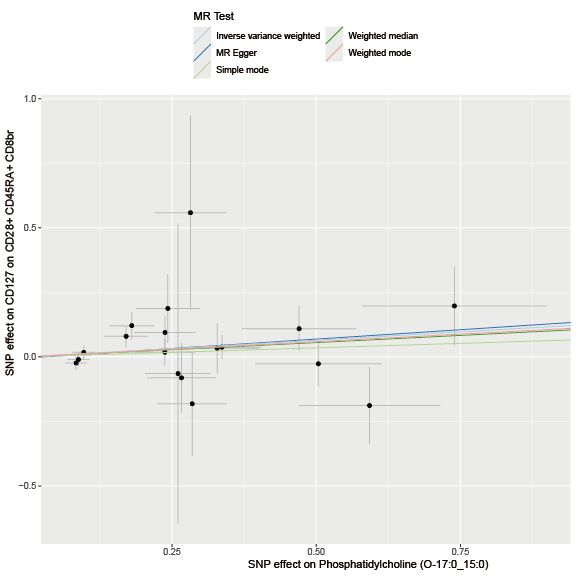
 Supplementary Figure 4C. Scatter plot (Phosphatidylcholine (O-17:0_15:0)-CD127 on CD28+ CD45RA+ CD8br)
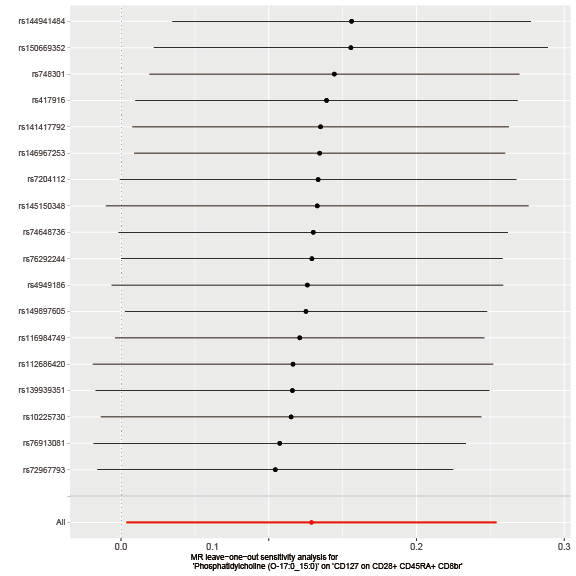
 Supplementary Figure 4D. Sensitivity-analysis (Phosphatidylcholine (O-17:0_15:0)-CD127 on CD28+ CD45RA+ CD8br)

## Supplementary Table 1. Sensitivity analysis between lipids and UC

| exposure | outcome | method | pval |
| --- | --- | --- | --- |
| Sterol ester (27:1/17:0) | UC | MR Egger/heterogeneity | 0.5231 |
|  |  | IVW/heterogeneity | 0.5652 |
|  |  | pleiotropy | 0.6197 |
| Sterol ester (27:1/18:1) | UC | MR Egger/heterogeneity | 0.0612 |
|  |  | IVW/heterogeneity | 0.0491 |
|  |  | pleiotropy | 0.2259 |
| Phosphatidylcholine (16:0_0:0) | UC | MR Egger/heterogeneity | 0.7637 |
|  |  | IVW/heterogeneity | 0.7724 |
|  |  | pleiotropy | 0.4039 |
| Phosphatidylcholine (20:4_0:0) | UC | MR Egger/heterogeneity | 0.5734 |
|  |  | IVW/heterogeneity | 0.5178 |
|  |  | pleiotropy | 0.1833 |
| Phosphatidylcholine (O-16:1_20:3) | UC | MR Egger/heterogeneity | 0.6259 |
|  |  | IVW/heterogeneity | 0.6189 |
|  |  | pleiotropy | 0.3118 |
| Phosphatidylcholine (O-17:0_15:0) | UC | MR Egger/heterogeneity | 0.9677 |
|  |  | IVW/heterogeneity | 0.9745 |
|  |  | pleiotropy | 0.5747 |

## Supplementary Table 2. Sensitivity analysis between lipids and immune cells

| exposure | Mediator | method | pval |
| --- | --- | --- | --- |
| Phosphatidylcholine (16:0_0:0) | Lymphocyte %leukocyte | MR Egger/heterogeneity | 0.5624 |
|  |  | IVW/heterogeneity | 0.6120 |
|  |  | pleiotropy | 0.7446 |
| Phosphatidylcholine (16:0_0:0) | CD11b on CD33+ HLA DR+ CD14dim | MR Egger/heterogeneity | 0.5918 |
|  |  | IVW/heterogeneity | 0.6433 |
|  |  | pleiotropy | 0.8079 |
| Phosphatidylcholine (20:4_0:0) | CD11b on Mo MDSC | MR Egger/heterogeneity | 0.8964 |
|  |  | IVW/heterogeneity | 0.9173 |
|  |  | pleiotropy | 0.6756 |
| Phosphatidylcholine (O-17:0_15:0) | CD127 on CD28+ CD45RA+ CD8br | MR Egger/heterogeneity | 0.2596 |
|  |  | IVW/heterogeneity | 0.3162 |
|  |  | pleiotropy | 0.8547 |
